# Invasive breast carcinoma in a patient with PHTS: a case report

**DOI:** 10.1186/s13000-025-01715-1

**Published:** 2025-10-17

**Authors:** Haiying Zhan, Neal Fischbach, Melanie Lynch, Yuanxin Liang, Uma Krishnamurti, Paul Cohen

**Affiliations:** 1 Department of Pathology, Yale University School of Medicine, New Haven, CT 06520 USA; 2 Department of Oncology, Yale University School of Medicine, New Haven, CT 06520 USA; 3 Department of Surgery, Yale University School of Medicine, New Haven, CT 06520 USA

## Abstract

**Background:**

PTEN hamartoma tumor syndrome (PHTS) is a rare, multisystem disorder caused by germline pathogenic variants in the PTEN gene, predisposing individuals to various malignancies, including breast cancer.

**Case presentation:**

We describe a 26-year-old woman with longstanding bilateral palpable breast masses and spontaneous bloody nipple discharge. Imaging revealed numerous cysts and masses, predominantly in the right breast. Multiple biopsies showed benign papilloma with focal atypical ductal hyperplasia (ADH), while total mastectomy specimens revealed multifocal, poorly differentiated, triple-negative invasive carcinoma. An axillary lymph node contained ectopic breast tissue with associated papillary proliferation. Genetic testing identified a pathogenic germline *PTEN* variant (c.209 + 4_209 + 7delAGTA), confirming PTEN hamartoma tumor syndrome (PHTS).

**Conclusion:**

This case underscores the importance of considering PHTS in young patients presenting with extensive papillomatosis and other unusual breast pathologic findings, even in the absence of a family history of cancer. Early recognition enables timely genetic counseling, confirmatory testing, and implementation of appropriate surveillance and management strategies.

## Introduction

PTEN hamartoma tumor syndrome (PHTS) is a heterogeneous, multisystem disorder caused by germline pathogenic variants (PVs) in the *PTEN* (*phosphatase and tensin homolog*) gene. Individuals with PHTS are predisposed to developing multiple hamartomatous lesions and have increased risks of various malignancies, including breast, thyroid, endometrium, kidney and colorectal cancers [1]. Cowden’s syndrome refers to a subset of PHTS characterized by increased risk of cancer, most commonly affecting breast, thyroid, and uterus. Women with Cowden’s syndrome have up to 85% lifetime risk of breast cancer, dramatically higher than that in the general population (12.9% lifetime risk) and approximating that of germline mutations in the BRCA1/2 genes [2]. On the other hand, papillomatosis without atypia is associated with very little increase in lifetime risk of breast cancer. Here, we present an unusual case of invasive breast carcinoma arising in a young woman with longstanding papillomatosis ultimately found related to a pathogenic germline *PTEN* mutation.

## Case presentation

### Clinical presentation

25-year-old women presented with bilateral palpable breast masses and spontaneous bloody nipple discharge, most prominent in the right breast, since 2016. She had no significant medical history or family history. She underwent right breast biopsy in 2018 with findings of atypical ductal hyperplasia (ADH) involving a complex fibroadenomatous and papillary lesion at the 2:00 position, and benign breast tissue with stromal fibrosis in the retroareolar region. More recently, she developed bilateral palpable breast masses and continuous bilateral bloody nipple discharge. She underwent right breast excisional biopsy in 2024 and subsequent right mastectomy and sentinel node biopsy.

### Imaging studies of bilateral breasts

**Fig. 1:**
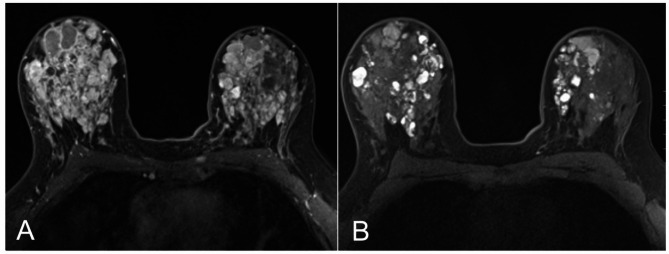
Ultrasound (**A**) and MRI (**B**) of bilateral breasts

Ultrasound imaging of bilateral breasts revealed multiple oval hypoechoic masses with circumscribed margins. In the right breast, lesions were identified in the retroareolar region as well as at 1 o’clock, 6 o’clock, and 9 o’clock, measuring up to 1.6 cm in greatest dimension (Fig. 1A). These masses had increased in size compared to imaging performed six months earlier and demonstrated no internal vascularity. Additionally, multiple circumscribed intraductal masses with associated internal vascularity were present. Persistent cystic duct ectasia was noted in the retroareolar region of the right breast, accompanied by intraductal thickening and papillary projections. The left breast showed a 1.5 cm oval mass lesion with circumscribed margin, and features of duct ectasia.

MRI of the breasts revealed innumerable cystic lesions throughout both breasts, some of which demonstrate fluid-fluid levels, including a 1.7 cm × 1.5 cm × 1.4 cm cyst at the 3 o’clock position of the right breast, likely representing a proteinaceous cyst. Numerous cystic structures in the bilateral retroareolar regions appeared interconnected, consistent with ductal ectasia (Fig. 1B). Additionally, prominent axillary lymph nodes were noted bilaterally, measuring up to 0.9 cm in short-axis on the right and up to 0.7 cm on the left.

### Pathological and molecular analysis

**Fig. 2:**
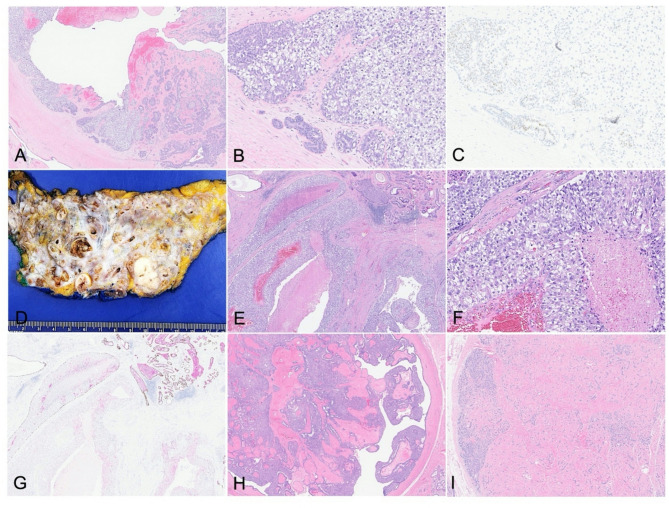
**A**-**C**: Excisional biopsy of the right breast showing high-grade clear cell type DCIS involving papilloma. (**A**) H&E image (4x). (**B**) H&E image (20x). (**C**) ER immunostaining. **D**-**I**: Right breast mastectomy showing multifocal poorly differentiated invasive carcinoma. (**D**) Gross examination of right breast mastectomy. (**E**) H&E image (4x). (**F**) H&E image (20x). (**G**) p40-CK5/6 dual immunostains demonstrated the lack of myoepithelial cells in the invasive carcinoma. (**H**) Background breast tissue showing papillomatosis and fibroadenomas (**I**)

The excisional biopsy revealed high-grade ductal carcinoma in situ (DCIS) with clear cell change (Fig. 2A and B), arising in a background of papilloma with florid usual ductal hyperplasia, fibroadenomatoid nodules, and cysts. Immunohistochemical staining for p40 highlighted the presence of myoepithelial cells both at the duct periphery and along the fibrovascular cores. The atypical epithelial cells were positive for estrogen receptor (ER) and negative for cytokeratin 5/6 (CK5/6) while the neighboring benign proliferation areas showed heterogenous CK5/6 and ER expression (Fig. 1C).

Subsequent germline genetic testing confirmed the presence of a pathogenic *PTEN* gene alteration: c.209 + 4_209 + 7delAGTA, located within intron 3. This 4-nucleotide deletion is consistent with *PTEN* hamartoma tumor syndrome (PHTS) and has been reported in multiple individuals exhibiting clinical features of Cowden syndrome [3, 4]. In addition, a variant of uncertain significance was identified in the NF1 gene: p.N295K (c.885T > A). No other deleterious germline mutations were detected.

The patient subsequently underwent right total mastectomy. Gross examination of the specimen showed diffusely nodular, cystic, and densely fibrous breast tissue. The cystic nodules contained variable contents, ranging from dark red clotted blood to tan-white pasty material. Tan-pink papillary excrescences were observed adherent to the walls of several locules. The cysts measured up to 1.5 cm in greatest dimension.

Microscopic examination revealed at least five foci of poorly differentiated invasive ductal carcinoma involving all four quadrants of the breast, with tumor foci measuring from 0.8 cm to 2.7 cm in greatest dimension (Fig. 2D). The invasive carcinoma was ER-negative, PR-negative, HER2 2+ (equivocal). Reflex HER2 in situ hybridization (FISH) was negative for amplification (HER2/CEP17 ratio: 1.9; average HER2 copy number: 2.8 signals per cell). Ki-67 proliferation index was markedly elevated at 80%. The background breast parenchyma displayed a complex combination of premalignant and benign changes, including numerous intraductal papillomas, some of which are involved by high-grade DCIS as well as florid usual ductal hyperplasia (Fig. 2E). Multiple fibroadenomas were also identified throughout the specimen (Fig. 2F). Invasive carcinoma extensively involved initial surgical margins, prompting additional chest wall excisions. Immunohistochemical studies demonstrated absence of myoepithelial cells surrounding the invasive foci, as highlighted by p40-CK5/6 dual staining (Fig. 2G) and smooth muscle myosin (SMM), supporting the diagnosis of invasive carcinoma. The tumor cells showed diffuse and strong nuclear p53 expression. Retinoblastoma (RB1) and SDHB protein expressions were retained (not shown).

**Fig. 3:**
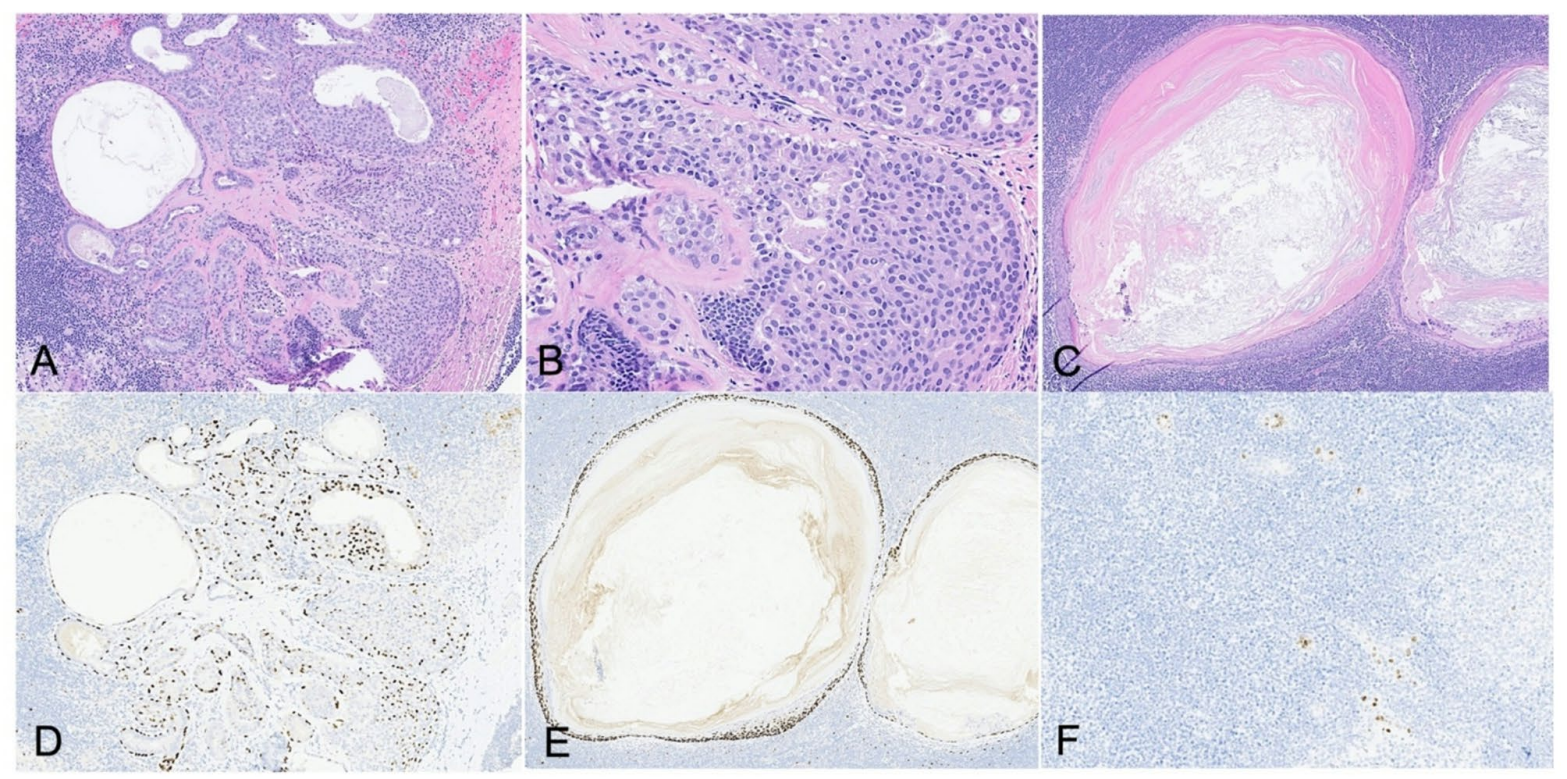
Axillary lymph node with intranodal papillary proliferation (**A**, **B**) and epidermoid inclusion cyst (**C**). (**D**-**E**) p63 immunotaining in papillary proliferation lesion and epidermoid inclusion cyst. (**F**) ER shows heterogenous immunostaining

One axillary lymph node showed an intranodal papillary proliferation and epidermoid inclusion cyst (Fig. 3A and C). Both p40 and p63 highlighted the presence of myoepithelial cells within the papillary proliferation (Fig. 3D), as well as the squamous component of the epidermoid inclusion cyst (Fig. 3E). CK5/6 and ER (Fig. 3F) immunostains showed heterogenous expression patterns. The combined histologic and immunohistochemical findings were consistent with a benign intranodal proliferative process.

### Treatment and follow up

Given the very high likelihood of invasive, triple negative breast cancer in the left breast, and significant risk of systemic recurrence from her right breast cancer, the patient initiated treatment with weekly carboplatin and paclitaxel followed by dose dense Adriamycin and cyclophosphamide with concurrent pembrolizumab as per the Keynote 522 regimen [5]. Ultimately, she will undergo left mastectomy and sentinel node biopsy.

## Discussion

There is strong evidence supporting an increased risk of breast cancer in women with germline pathogenic variants (PVs) in the *PTEN* gene [2, 6, 7]. Available studies estimate the lifetime risk of breast cancer in individuals with PTEN hamartoma tumor syndrome to be between 67 and 85%, a risk comparable to that observed in carriers of germline *BRCA1* or *BRCA2* mutations. Breast cancers arising in the setting of PHTS are clinically distinct from sporadic BCs, including younger age at onset, multifocality, and a higher risk of developing second primary breast cancers. Among 44 reported PHTS-associated breast cancers, the majority exhibited ductal histology (86.4%) and were positive for estrogen receptor (84.1%) and progesterone receptor (81.8%), and negative for HER2 (84.1%). Most tumor were moderately differentiated (52.3%) [8]. Additionally, breast cancers from PHTS patients harboring germline pathogenic or likely pathogenic *PTEN* variants demonstrated significantly fewer somatic *PIK3CA* mutations compared to those reported in The Cancer Genome Atlas (TCGA) cohort and PHTS-associated breast cancers with *PTEN* variants of uncertain significance or likely benign PTEN variants [8].

This case highlighted a young patient with a confirmed germline *PTEN* mutation, initially presenting with clinical features suggestive of papillomatosis with focal atypia. However, subsequent excisional biopsy and mastectomy revealed multifocal, poorly differentiated, triple-negative breast cancer (TNBC), staged as pT2(m)N0(sn)M0, arising in a background of extensive papillomatosis and fibroadenomas. Both the invasive and in situ carcinoma components displayed prominent clear cell features and diffuse p53 overexpression. Notably, individuals with germline *PTEN* mutations have also been reported to carry variants in succinate dehydrogenase complex subunit (SDHx) genes [9, 10]. In this case, tumor cells showed retained SDHB immunostaining.

This case also demonstrated benign papillary proliferation of ectopic breast tissue (EBT) within axillary lymph nodes, mirroring the diffuse papillomatosis observed throughout the breast parenchyma. In this specific context, the intranodal papillary proliferation was likely related to the underlying *PTEN* mutation. While the EBT in axillary lymph nodes is a benign condition, it can undergo proliferative changes—including intraductal papilloma (IDP)—and may rarely exhibit features of atypical ductal hyperplasia (ADH) or ductal carcinoma in situ (DCIS) [11, 12]. These intranodal lesions are generally believed to arise independently as de novo proliferations within lymph nodes and are most often associated with papillary and other noninvasive breast neoplasms. Although rare, intranodal papillary proliferations may be linked to *PTEN* mutations, as demonstrated in this case. From a diagnostic standpoint, distinctive morphological features—such as the characteristic two-cell pattern—together with a panel of immunohistochemical stains including AE1/AE3, alpha-smooth muscle actin, p63, ER, and CK5/6—facilitate reliable differentiation of benign ectopic IDP from atypical or malignant lesions. Accurate diagnosis is critical to prevent overtreatment and support optimal patient care.

Cowden’s syndrome is a critical subset of PHTS which is vital to recognize due to its well-recognized association with breast, endometrial, thyroid, and other cancers. However, the *PTEN* mutations observed in Cowden’s syndrome are not specific for Cowden’s syndrome versus other subsets of PHTS. Intraductal papilloma and other proliferative breast lesions are a common feature of Cowden’s syndrome, though not included in the diagnostic criteria outlined [13]. Given the syndrome’s rarity, lack of family history of malignancy in most affected women, and the variable syndrome manifestations, a high index of suspicion for deleterious mutations in *PTEN* is critical for appropriate referral for genetic counseling and testing. At the molecular level, loss of PTEN function results in aberrant activation of the PI3K/AKT/mTOR signaling pathway. As such, the AKT1-inhibitor capivasertib is now FDA approved for patients with ER-positive metastatic breast cancer and AKT pathway mutations [14]. Similarly, the mTOR inhibitor, everolimus, also FDA approved for the treatment of metastatic ER + breast cancer, may be more active in certain subsets of women with AKT-pathway mutations [15]. At present, there is no proven role for AKT-pathway inhibitors in the adjuvant setting. Women with Cowden’s syndrome are also at elevated risk for other malignancies, including endometrial, thyroid, renal, and colorectal cancers [9, 10]. Accordingly, comprehensive surveillance for these associated malignancies is strongly recommended, along with predictive genetic testing for first-degree relatives.

## Data Availability

The imaging and pathologic data are available from the corresponding author on reasonable request.
